# Scientific Items

**Published:** 1887-03

**Authors:** 


### SCIENTIFIC ITEMS.

**The Atmosphere of Caves.**—The largest volume of subterranean atmosphere with which we are acquainted is found in Mammoth Cave, Ky. This cave, or rather system of caves, is very extensive, greatly exceeding the other two notable caverns in our country: the Luray of Virginia and the Wyandott of Southern Indiana. The passageways of Mammoth Cave have a combined length of over 150 miles, and their area covers hundreds of acres. It is estimated that the entire volume of atmosphere thus inclosed exceeds twelve million cubic yards. In this underground world the ordinary atmospheric changes are unknown, summer and winter are unknown, and the heat of the sun never reaches its unbroken night.

Like all our larger caverns, the temperature is alike at all times and seasons. In summer there is a brisk outward current, having a temperature of 53° to 54°. This current is doubtless fed by certain leakages of air which filter through the sinkholes, which discharge their moisture at certain points in the cave system.

In the winter there is a current of air setting inward. This is not perceived at a distance of one-fourth of a mile from the entrance. It nevertheless depresses the thermometer a few degrees, and its effect upon the humidity of the air is evident at the distance of three-fourths of a mile.

For the first time hygrometric observations have been carefully made as to this unique body of atmosphere. The dryness of the air has often been noticed, and the resultant nitre beds were esteemed a matter of national importance during the war of 1812.

Several consumptives once tried to live, and get well, in the cave. But the result was disastrous. The lack of light was, no doubt, one reason of this; but the hygrometric condition of the air, of which nothing was then known, by greatly retarding healthy perspiration, doubtless hastened the fatal result.

**Meteor Showers.**—Prof. Richard A. Proctor maintains that most of the meteor streams with which the earth comes in contact are derived from the earth itself; that is, thrown off by volcanic action at a time when the internal forces of our planet were sufficiently active to give the initial velocity, some twelve miles a second, requisite to carry them beyond the earth's attraction. Comets, which he regards as the parents of the meteor streams, he thinks may have originated outside our solar system. Most of the comets whose orbits belong to our system, he thinks originated in the larger planets. The sun is now, perhaps, giving birth frequently to comets which probably pass beyond the limits of its attraction.—*Ex.*

**The Lick Telescope.**—Among other large and noble benefactions, James Lick, the California millionaire, a few years ago, devoted a generous sum of money to the erection of a gigantic telescope.

This telescope, which will soon be placed in the observatory on the summit of Mount Hamilton, near San Francisco, is the largest of its kind which has ever been constructed on the earth, and, if successfully completed, will undoubtedly reveal to the human eye for the first time many a secret of the sky.

Some idea of the size and power of the Lick telescope may be gathered from the fact that it has a focus fifty-five feet long—nearly fifteen feet longer than the longest focus hitherto made. Like all modern telescopes, moreover, it is a refractor—that is, it will form the image directly to the eye.

The most important and interesting of all the parts of telescopes are their lenses. The lens is formed of two discs of glass, one convex and the other concave. The discs of the Lick lens are no less than three feet.

**Cementation.**—Some ingenious person, who recognizes that the present manner of disposing of the human body after death must be improved upon, has proposed a new method. He has applied for patents on the moulds, cements, etc., for performing what he terms "cementation." The body is to be encased in a cement which becomes as hard as stone and preserves the body forever. This certainly comes very near the old Egyptian practice of mummyfying. Before the advantages claimed over cremation are considered, he must prove that a cement can be made which is perfectly impenetrable to all gases and moisture. And even if this is shown, we think it is much better to destroy all useless organic matter, capable of decomposition and production of disease, than to cement it for generations to come.—*National Druggist.*

**Insects as Authors of Epidemics.**—Dr. R. L. Maddox, in a paper read before the Royal Microscopical Society, details the results of further experiments in feeding insects, especially the common blow-fly, on the comma bacillus. His observations include a large number of microscopical determinations. The results of all his investigations lead him to believe that the comma bacillus from cultures can pass in a living state through the digestive tubes of some insects, and, through this fact, that such insects are likely to become an important means of distributing disease, especially to animals that feed upon them. This is in accordance with the views of Dr. Grossi, that "insects, especially flies, may be considered as veritable authors of epidemics and agents in infectious maladies."—*Ex.*

**What of Ghosts?**—The editor of *Popular Science News* says: "The number of instances of alleged ghostly appearances and disturbance which have been brought to my knowledge by parties influenced by a sincere desire to learn the cause and nature of the phenomena, is quite large, and extend over a period of half a century. In reviewing these occurrences late in life, after many years devoted to practical scientific investigation, I fail to find but few which do not present problems and difficulties inexplicable upon any hypothesis based on natural laws as at present understood.

